# Fatty acid synthase may facilitate the trafficking of bovine alpha herpesvirus 1 out of the Golgi apparatus, potentially promoting viral infection

**DOI:** 10.1128/spectrum.01388-25

**Published:** 2025-11-06

**Authors:** Xiaozhen Ma, Wenyuan Gu, Xuan Li, Xiuyan Ding, Liqian Zhu

**Affiliations:** 1 College of Life Sciences, Hebei University 56667, Baoding, China; 2 Center for Animal Diseases Control and Prevention of Hebei Province, Shijiazhuang, China; University of Florida College of Dentistry, Gainesville, Florida, USA

**Keywords:** BoAHV1, FASN, viral replication

## Abstract

The lipogenic enzyme fatty acid synthase (FASN) is essential for the *de novo* synthesis of fatty acids, and its aberrant expression has been implicated in the development of various diseases. However, the interaction between BoAHV-1 infection and FASN remains to be fully elucidated. In this study, we observed that both viral acute infection and latency in bovine trigeminal ganglia (TG) neurons, as well as virus productive infection at later stages in Neuro-2A and MDBK cells, significantly reduced FASN protein levels. Notably, a subset of FASN protein co-localized with the viral glycoprotein gD, as revealed by confocal microscopy in these cell cultures. Knockdown of FASN protein expression using siRNAs or chemical inhibition of FASN by Cerulenin leads to reduced viral replication. Interestingly, a subset of FASN proteins localizes in the Golgi apparatus, and Cerulenin treatment retards the trafficking of virions out of the Golgi. Collectively, our findings reveal a novel mechanism by which FASN regulates viral replication: a portion of the FASN protein located in the Golgi apparatus facilitates viral trafficking out of the Golgi, which is essential for the completion of the viral replication cycle.

**IMPORTANCE:**

Here, we found that fatty acid synthase (FASN) protein is potentially involved in virus infection both *in vitro* and *in vivo*. In terms of mechanism, a subset of FASN protein co-localizes with the viral glycoprotein gD, as revealed by confocal microscopy in both MDBK and Neuro-2A cell cultures. Interestingly, a subset of FASN protein localizes in the Golgi apparatus, and Cerulenin treatment retards the trafficking of virions out of the Golgi. Therefore, we propose that a portion of the FASN protein in the Golgi apparatus plays an important role in viral trafficking out of the Golgi, which is essential for the completion of the viral replication cycle. This represents a novel mechanism of virus infection regulation, which has not been reported in other viruses, although the FASN protein is involved in the regulation of multiple viruses through distinct mechanisms.

## INTRODUCTION

Bovine alpha herpesvirus 1 (BoAHV-1) is a neurotropic virus that belongs to the genus *Varicellovirus* in the subfamily *Alphaherpesvirinae* under the family *Herpesviridae* (1–3). After the initial mucosal infection in the oral, nasal, and ocular cavities, BoAHV-1 is transported to sensory neurons within the trigeminal ganglia (TG), which serves as a primary site for lifelong latency reservoirs. Here, the latency can be reactivated by various stressors, resulting in viral shedding and the progression to disease (4, 5). The virus infection or latency-reactivation may commonly induce bovine infectious rhinotracheitis (IBR), conjunctivitis, vulvovaginitis, meningoencephalitis, and abortion, causing significant economic losses to the cattle industry (3, 6, 7). The virus is currently impacting the cattle industry worldwide. Reports indicate that it inflicts approximately $3 billion in annual losses on the US cattle industry (8).

The enzyme fatty acid synthase (FASN) is a multifunctional protein that primarily catalyzes the conversion of acetyl-CoA and malonyl-CoA into palmitate, using NADPH as a reducing agent (9, 10). This process is essential for the production of cellular lipids, including triglycerides and phospholipids. In addition to its role in fatty acid synthesis, abnormal expression of FASN is implicated in various diseases, such as metabolic disorders, cancers (9, 11, 12), and inflammatory diseases (13, 14). As a result, FASN has been recognized as a potential therapeutic target for cancer treatment.

Fatty acids are important energy sources, primarily catabolized by fatty acid β-oxidation (FAO) in mitochondria. Given that FAO produces more than three times the amount of ATP per mole compared to glucose oxidation, it serves as the preferred energy source for highly metabolized cells under physiological conditions (15). Carnitine palmitoyl-transferase 1 A (CPT1A) is the rate-limiting enzyme of FAO (16). Both FASN and CPT1A are thus key enzymes involved in lipid metabolism: FASN synthesizes fatty acids, while CPT1A transports them into mitochondria for oxidation. We have previously reported that BoAHV-1 productive infection affects CPT1A protein expression and localization, a potential mechanism to alter FAO (17). This prompted us to investigate whether and how FASN is involved in BoAHV-1 replication.

In this study, we examined the effects of BoAHV-1 infection on FASN protein expression both *in vivo* and *in vitro* and assessed its impact on viral productive infection in cell culture using siRNAs and chemical inhibitors. Our results demonstrate that FASN is involved in BoAHV-1 infection both *in vivo* and *in vitro*. For the first time, we have identified a novel mechanism by which a portion of the FASN protein in the Golgi apparatus is required in viral trafficking out of the Golgi. Our findings will extend our understanding of the viral replication mechanisms involving FASN molecules.

## RESULTS

### Detection of FASN protein expression in bovine TG neurons during viral acute infection

**Fig 1:**
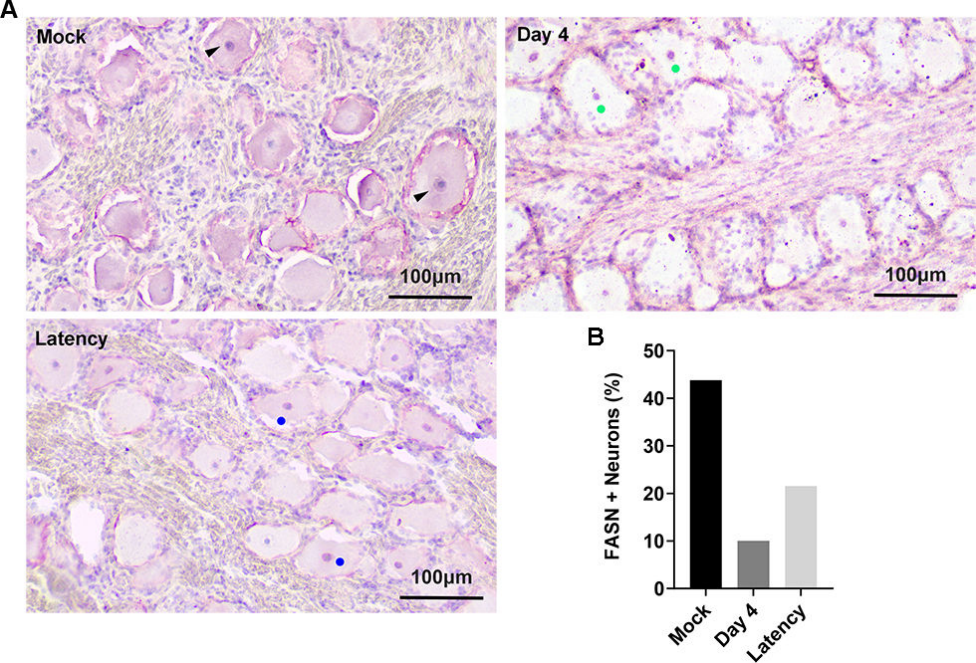
Detection of FASN protein in TG neurons during viral acute infection and latency by IHC. (**A**) TG tissues were collected from calves subjected to mock infection, acute infection (at 4 days post-infection), or latent infection (at 60 days post-infection). Thin sections of 10 µm were cut from formalin-fixed, paraffin-embedded TG tissues and subjected to FASN protein detection by IHC using FASN-specific polyclonal antibody (Proteintech, cat#10624-2-AP, 1:500). Biotinylated goat anti-rabbit IgG (Vector Laboratories) was used as the secondary antibody. (**B**) The percentage of FASN-positive neurons was calculated from 468 neurons of uninfected calves, 467 neurons from acutely infected calves, and 413 TG neurons from latency calves. Data shown are representative of two independent experiments. Scale bars = 100 µm.

To assess the involvement of the FASN protein in BoAHV-1 infection *in vivo*, healthy calves that tested negative for BoAHV-1 antibodies were used for the viral infection assay. Trigeminal ganglia (TG) tissues were harvested at 4 and 60 days post-infection, representing the acute phase and latency of viral infection, respectively, as previously described (18). Immunohistochemistry (IHC) was employed to evaluate the changes in FASN protein expression in response to viral infection in these TG neurons. As shown in Fig. 1A, extensive staining of FASN protein (denoted by black arrows) was readily detected in a subset of TG neurons from mock-infected calves, particularly in the cytoplasm. Meanwhile, moderate staining of FASN (denoted by filled blue circles) was readily detected in latently infected TG neurons. In contrast, faint staining was observed in the cytoplasm of TG neurons from BoAHV-1 acutely infected calves (Fig. 1A). Interestingly, FASN+ nuclei (denoted by filled green/blue circles) were readily observed in neurons from both acutely infected and latent cows. Quantitative analysis revealed that the proportion of FASN+ neurons, including both cytoplasmic and nuclear staining, was 43.80%, 10.06%, and 21.55% in calves with mock infection, acute infection, and latency, respectively (Fig. 1B). Although the proportion of FASN+ staining in latently infected TG neurons was higher than that in acutely infected calves, it was still lower than that in mock-infected neurons. These observations suggest that both acute and latent BoAHV-1 infection lead to a reduction in FASN protein expression in TG neurons, albeit to different extents. This indicates that FASN may play a role in the viral infection process.

### BoAHV-1 infection decreased FANS steady-state protein expression in cell cultures

**Fig 2:**
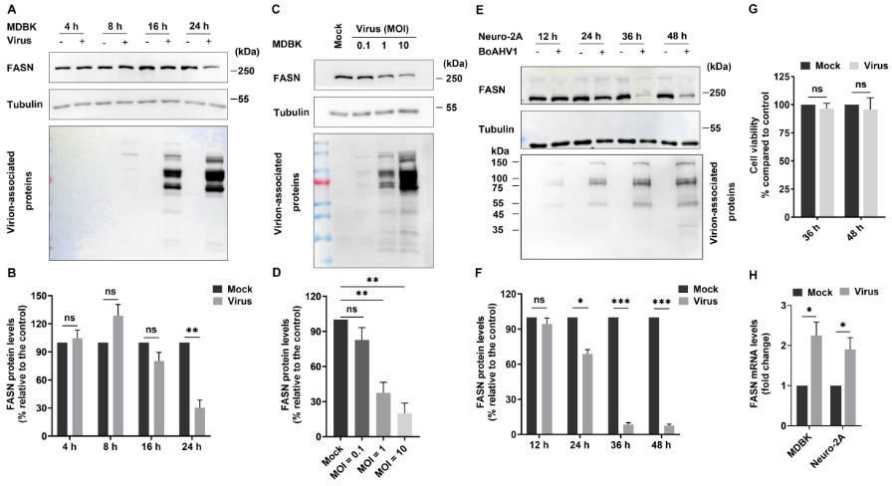
BoAHV-1 infection decreases the expression of FASN protein across various cell cultures. (**A**) MDBK cells that were confluent in 60 mm dishes were either mock infected or infected with BoAHV-1 at an MOI of 1. After 4, 8, 16, and 24 h of infection, cell lysates were prepared and analyzed by Western blotting using antibodies against FASN (Proteintech, cat# 10624-2-AP, 1:10,000) and virion-associated proteins (VMRD, cat# P170703-001, 1:5,000). (**C**) MDBK cells were either mock-infected or infected with BoAHV-1 at MOIs of 0.1, 1, and 10, respectively. At 24 hpi, cell lysates were prepared for Western blotting to assess the protein expression of FASN and virion-associated proteins. (**E**) Neuro-2A cells were either mock-infected or infected with BoAHV-1 at an MOI of 10. After 12, 24, 36, and 48 hours of infection, cell lysates were prepared and analyzed by Western blotting to detect FASN protein and virion-associated proteins. Tubulin was probed and served as a protein loading control and for subsequent quantitative analysis. (**B, D, and F**) Quantitative analysis of FASN band intensities was performed using the freeware software Image J. Changes in FASN protein levels after infection were calculated relative to mock-infected controls, which were set at 100%. (**G**) Neuro-2A cells in 24-well plates were either mock-infected or infected with BoAHV-1 at an MOI of 10. At 36 and 48 hpi, cell survival was assessed using the CCK-8 assay kit (Beyotime, Shanghai, China, cat# C0038), following the manufacturer’s instructions. (**H**) MDBK and Neuro-2A cells in six-well plates were either mock infected or infected with the virus (MOI of 1 for MDBK cells and MOI of 10 for Neuro-2A cells) for 24 h. Total RNA was then extracted from the cells for the detection of FASN mRNA using RT-qPCR. The data shown are means of three independent experiments with error bars indicating standard deviations. Significance was assessed by standard *t*-test (ns, not significant; *P < 0.05; **P  <  0.01; ***P  <  0.001).

Both bovine kidney (MDBK) and mouse neuroblastoma cells (Neuro-2A) were employed to investigate the effects of viral productive infection had on FASN protein expression *in vitro*. MDBK cells support the virus productive infection with high efficacy. BoAHV-1 can infect Neuro-2A cells, albeit with lower efficiency (19–21). When MDBK cells were infected with BoAHV-1 at a multiplicity of infection (MOI) of 1 over various time points, FASN protein levels significantly decreased at 24 hpi, showing a reduction of approximately 30.48% compared to the mock-infected control (Fig. 2A and B). When MDBK cells were infected with BoAHV-1 at increasing MOIs ranging from 0.1 to 10 for 24 h, respectively, FASN protein levels gradually decreased, to an extent correlated with increasing MOIs (Fig. 2C). Quantitative analysis indicated that the protein levels reduced to approximately 37.46% and 20.09% following infection with MOI of 1 and 10, respectively (Fig. 2D). The viral infection-induced depletion of FASN was also observed in Neuro-2A cells (Fig. 2E). Compared to the control, FASN protein levels decreased to approximately 68.85%, 8.42%, and 7.6% after infection for 24, 36, and 48 h, respectively (Fig. 2F). Of note, viruses grow poorly in Neuro-2A cells (21). The dramatic depletion of FASN protein in Neuro-2A cells induced by viral infection was not due to virus-induced cell death, as determined by the CCK-8 assay (Fig. 2G). FASN mRNA levels increased approximately 2.25-fold in MDBK cells and 1.90-fold in Neuro-2A cells following virus infection for 24 h, relative to mock-infected controls (Fig. 2H). The decreased steady-state protein levels of FASN do not corroborate the increased mRNA levels in response to viral infection. The decreased FASN protein expression observed during BoAHV-1 productive infection in both MDBK and Neuro-2A cells is consistent with that observed in TG neurons during viral lytic infection.

### A portion of FASN co-localizes with the viral glycoprotein gD

**Fig 3:**
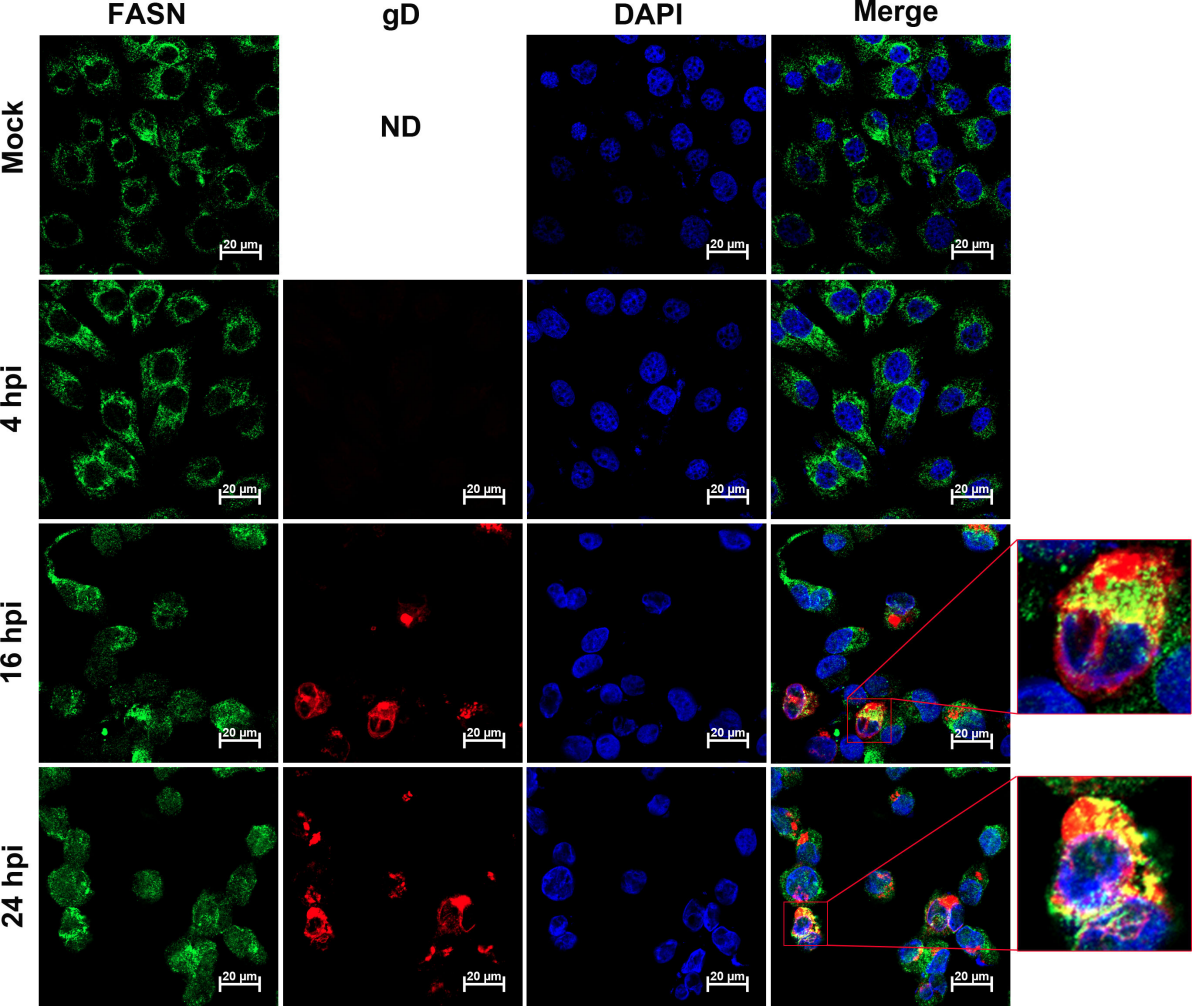
Examination of FASN localization in BoAHV-1-infected MDBK cells using IFA assay. MDBK cells were seeded into 24-well plates containing coverslips and cultured until they reached 90% confluence. The cells were then either mock-infected or infected with BoAHV-1 at an MOI of 1 for 4, 16, and 24 h, respectively. The cells were immunostained with antibodies against FASN (Green; Abclonal, cat# A21182, 1:200) and the viral protein gD (Red; VMRD, cat# 1B8-F11, 1:1,000). Nuclei were counterstained with DAPI (4′,6-diamidino-2-phenylindole; Blue). Immunofluorescence was visualized, and images were captured using confocal microscopy (Zeiss). Zoomed-in images framed in red highlight the typical co-localization of FASN with the viral protein gD. These images are representative of results from three independent experiments. Scale bars = 20 µm. ND, not done.

**Fig 4:**
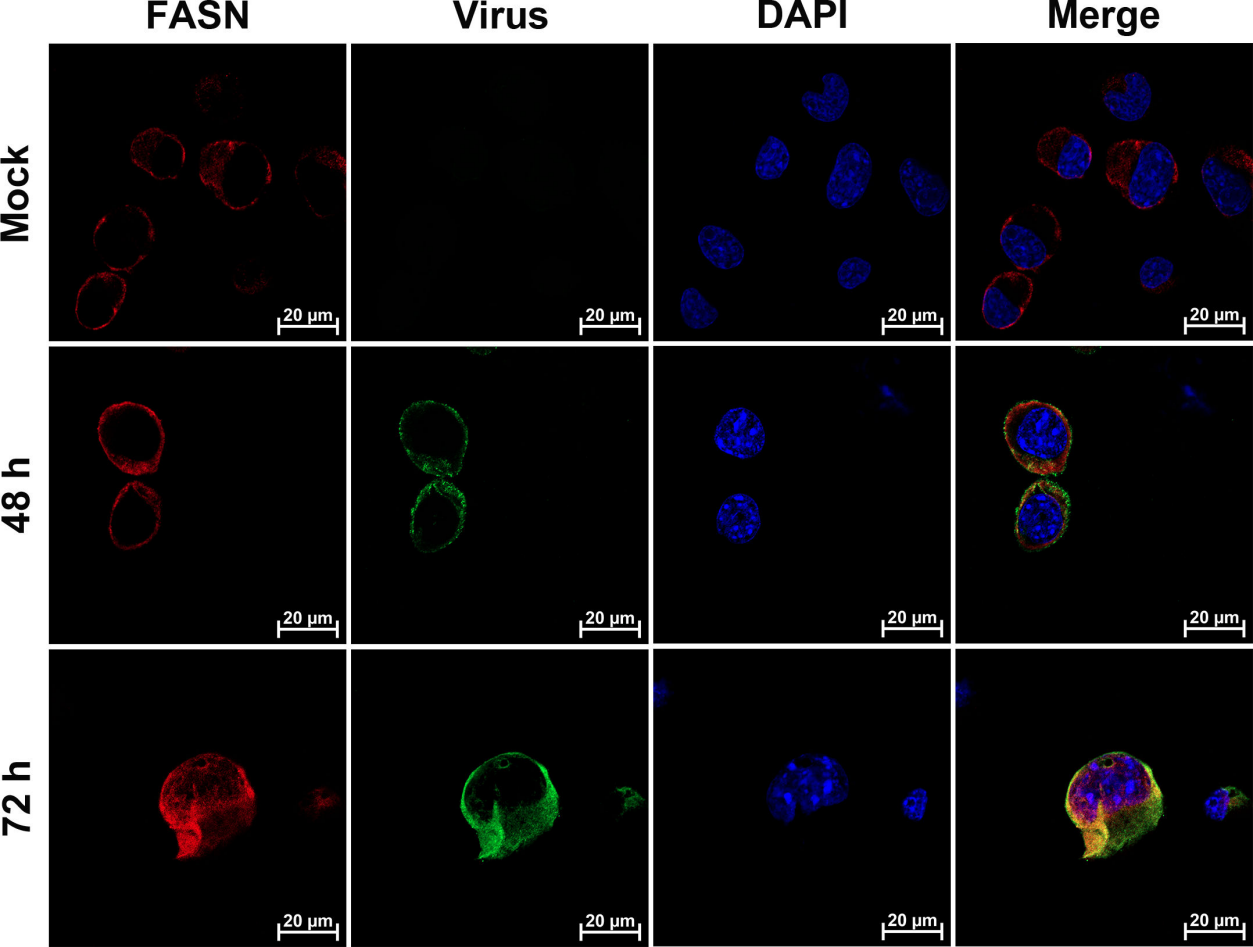
Examination of FASN localization in BoAHV-1-infected Neuro-2A cells using IFA assay. Neuro-2A cells were seeded into 24-well plates containing coverslips and cultured until they reached 90% confluence. The cells were then either mock-infected or infected with BoAHV-1 at an MOI of 1 for 48 and 72 h, respectively. They were immunostained with antibodies against the FASN protein (Red; Proteintech, cat# 10624-2-AP, 1:600) and the virion-associated protein (Green; VMRD, cat# P170703-001, 1:2,000). Nuclei were counterstained with DAPI (Blue). Immunofluorescence was visualized, and images were captured using confocal microscopy (Zeiss). These images are representative of results from three independent experiments. Scale bars = 20 µm.

An immunofluorescence assay (IFA) was performed using antibodies against FASN and the viral protein gD to determine whether BoAHV-1 infection affects FASN localization *in vitro*. Time points of 4, 16, and 24 hpi in MDBK cells were selected for this analysis. As a result, FASN was readily detected in the cytosol in mock-infected cells. At 4 hpi, FASN exhibited a localization pattern similar to that of the mock-infected control. However, at 16 and 24 hpi, re-localization of FASN was observed, with a subset of FASN showing highlighted staining forming irregular shapes in the cytoplasm. Interestingly, FASN co-localized well with the viral protein gD in virus-infected MDBK cells at both 16 and 24 hpi (Fig. 3). Similarly, co-localization of virion-associated protein with FASN was observed in the cytoplasm of virus-infected Neuro-2A cells at 48 and 72 hpi (Fig. 4). Although the overall localization of FASN was not dramatically altered in these cells, as seen in virus-infected MDBK cells, these findings suggest that FASN proteins are redistributed to areas overlapping or in close proximity to sites containing viral protein gD or virions.

### A subset of FASN is located in the Golgi apparatus, and its content is reduced following BoAHV-1 productive infection

**Fig 5:**
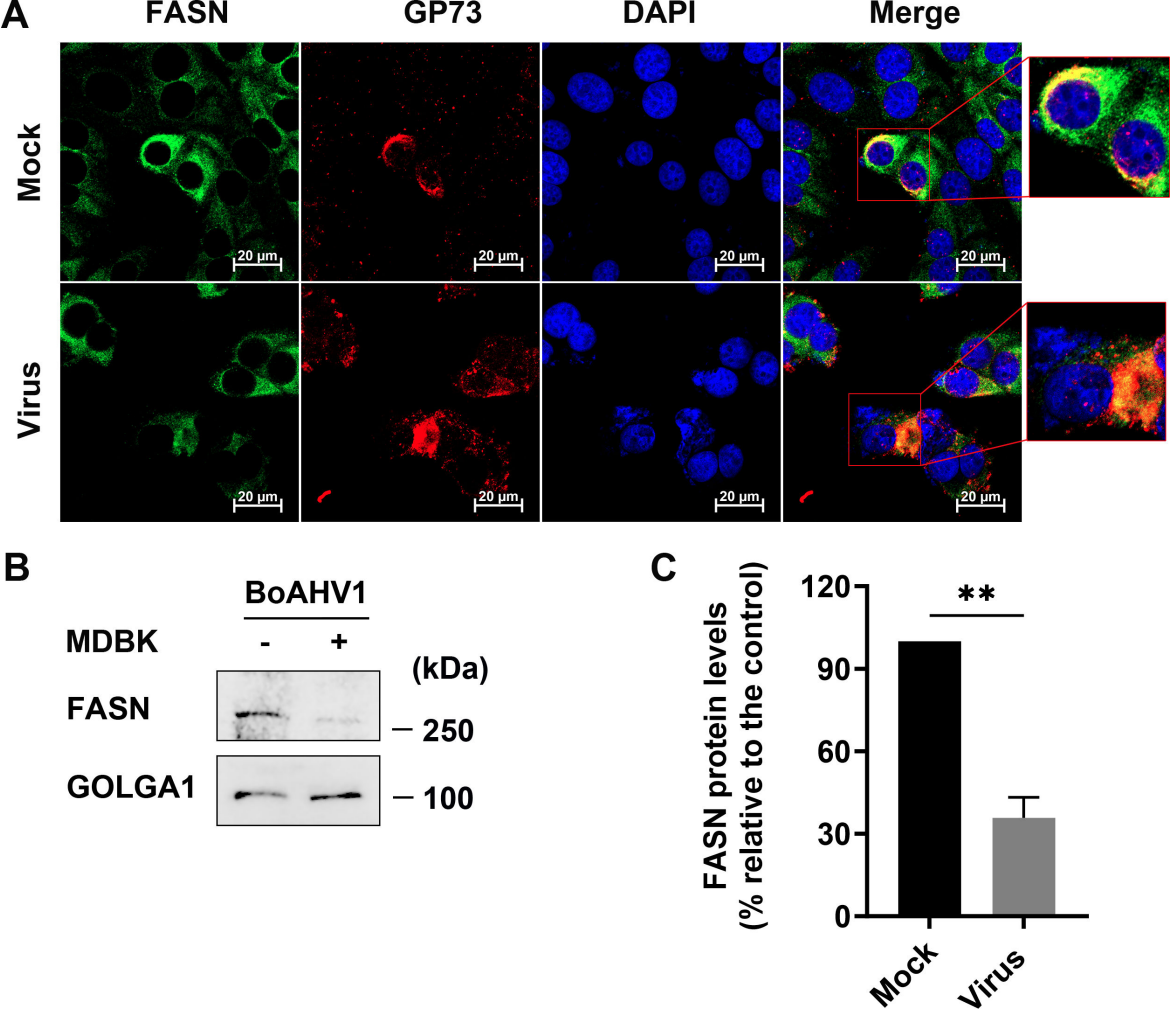
Examination of FASN protein in the Golgi apparatus in MDBK cells. (**A**) MDBK cells were seeded into 24-well plates containing coverslips and cultured until they reached 90% confluence. The cells were then either mock-infected or infected with BoAHV-1 at an MOI of 1 for 24 h. They were immunostained with antibodies against the FASN protein (Green; Proteintech, cat# 10624-2-AP, 1:600) and GP73 (Red; Proteintech, cat# 66331-1-1g, 1:200). Nuclei were counterstained with DAPI (Blue). Immunofluorescence was visualized, and images were captured using confocal microscopy (Zeiss). These images are representative of results from three independent experiments. Scale bars = 20 µm. (**B**) MDBK cells in 100 mm dishes were either mock-infected or infected with BoAHV-1 (MOI = 1) for 24 h. The cells were then collected to isolate the Golgi fraction using a commercial kit (Beijing Biolabo Technology, cat# HR0247-50T), following the manufacturer’s protocol. The lysates were subjected to Western blot analysis using antibodies against FASN (Proteintech, cat# 10624-2-AP, 1:10,000) and GOLGA1 (Abclonal, cat# A14688, 1:1,000). GOLGA1 served as a marker for the Golgi apparatus and as a protein loading control. The data presented are representative of three independent experiments. (**C**) Quantitative analysis of FASN band intensities was performed using the freeware software Image J. Changes in FASN protein levels after infection were calculated relative to mock-infected controls, which were set at 100%. The data shown are means of three independent experiments with error bars indicating standard deviations. Significance was assessed by a standard *t*-test (**P < 0.01).

The distribution profile of FASN proteins induced by viral infection appears similar to that of the Golgi apparatus, as we have previously reported (22). To determine whether a subset of FASN proteins accumulates in the Golgi apparatus, IFA was performed to detect FASN and the Golgi marker protein GP73 in the same cells. We found that a subset of FASN protein co-localized well with the Golgi marker protein GP73, regardless of viral infection, indicating its accumulation in the Golgi apparatus (Fig. 5A, zoom-in areas).

Next, the Golgi apparatus fractions were isolated from MDBK cells using a commercial Golgi purification kit and subjected to Western blot analysis to probe FASN protein. The Golgi marker protein GOLGA1 was used as a loading control. Our results indicated that FASN protein was readily detected in the Golgi fractions, regardless of viral infection. However, its accumulation in the Golgi fractions was significantly reduced following viral infection (Fig. 5B). Quantitative analysis indicated that the Golgi accumulated FASN protein levels decreased to approximately 35.75%, following virus infection (Fig. 5C).

Thus, using two independent methods, we demonstrated that a portion of FASN protein localizes in the Golgi apparatus, and its content is reduced following viral infection. These findings further support our findings that viral productive infection leads to the re-localization of FASN protein.

### FASN plays an important role in BoAHV-1 productive infection in both MDBK and Neuro-2A cells

**Fig 6:**
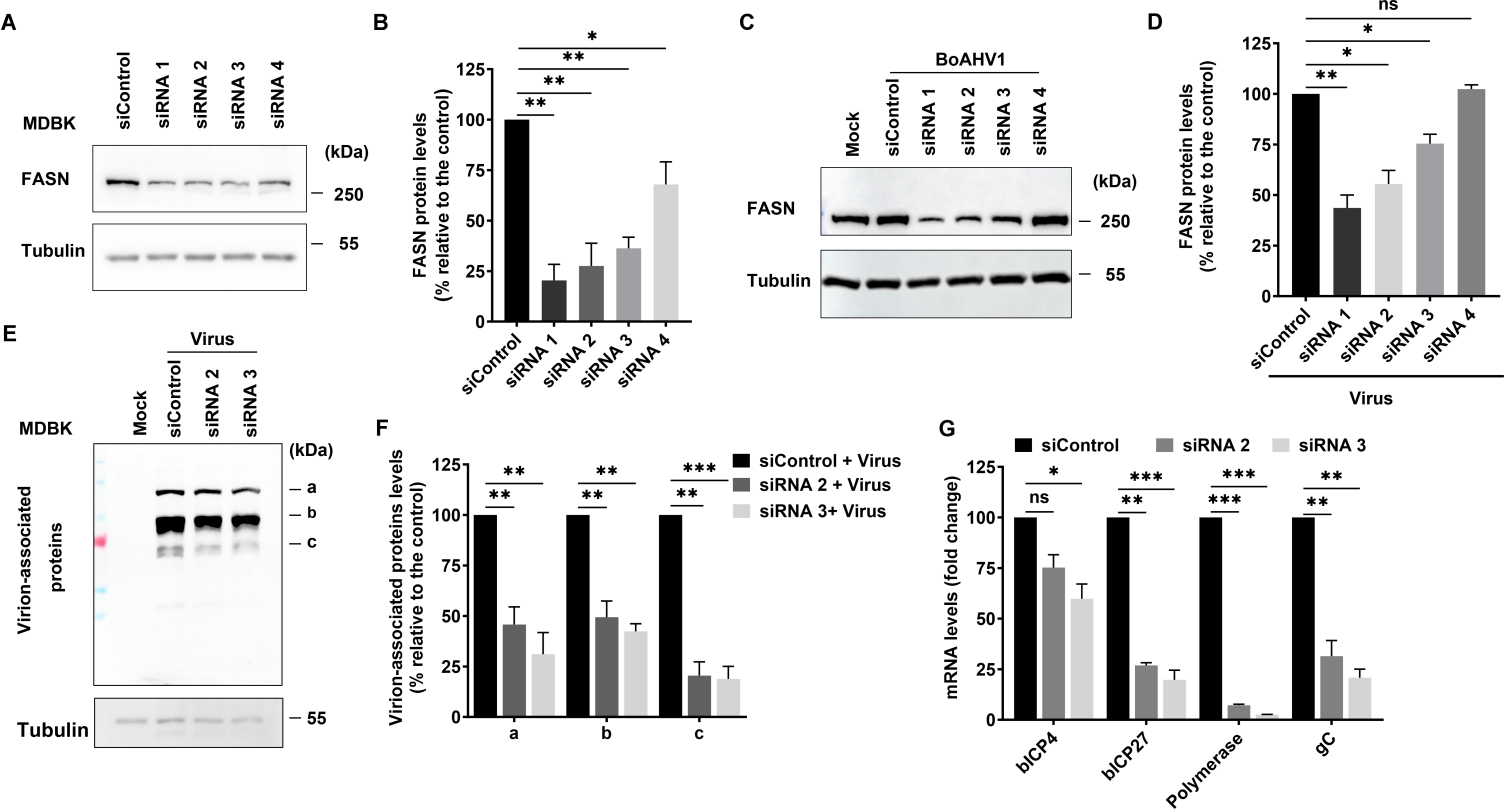
Determine the roles of FASN in BoAHV-1 productive infection in MDBK cells using transfection of siRNAs. (**A**) MDBK cells in six-well plates were transfected with either scrambled siRNA (150 pmol) or individual siRNAs targeting FASN (150 pmol), referred to as siRNA1, siRNA2, siRNA3, and siRNA4, respectively. At 48 h after transfection, cell lysates were prepared and subjected to Western blot analysis to detect FASN protein levels using an antibody against FASN (Proteintech, cat# 10624-2-AP, 1:10,000). (**C and E**) MDBK cells in six-well plates were transfected with either scrambled siRNA (150 pmol) or the indicated siRNAs (150 pmol). At 36 hpi, the cells were infected with BoAHV-1 at an MOI of 1. After 24 h of infection, the cells were collected and subjected to Western blot analysis using antibodies against FASN (Proteintech, cat# 10624-2-AP, 1:10,000) (**C**) and virus-associated proteins (VMRD, cat# P170703-001, 1:5,000) (**E**). (**B, D, and F**) The band intensities were quantified using the free software Image J. The intensity of each band was first normalized to that of the respective loading control Tubulin, and then normalized to that of the control transfected with scramble siRNA, which was arbitrarily set to 100%. (**G**) MDBK cells in six-well plates were transfected with either scrambled siRNA (150 pmol) or the indicated siRNAs (150 pmol). At 36 h post-transfection, the cells were infected with the virus at an MOI of 1 for 24 h. Subsequently, total RNA was extracted from the cells for the detection of viral mRNA using RT-qPCR. Primers specific for bICP4, bICP27, viral DNA polymerase, and viral protein gC were used, respectively. The data shown are means of three independent experiments, with error bars representing standard deviations. Significance was assessed using a Student’s *t*-test (ns, not significant; *P < 0.05; **P  <  0.01; ***P  <  0.001).

The roles of FASN in BoAHV-1 productive infection were analyzed in MDBK cells using FASN-specific siRNAs. Four commercially available siRNAs, designated as siRNA1, siRNA2, siRNA3, and siRNA4, could effectively reduce FASN protein levels to varying extents (Fig. 6A). FASN protein levels were reduced to approximately 20.41%, 27.54%, 36.29%, and 67.93% by these four individual siRNAs, respectively, compared to the scrambled siRNA control (Fig. 6B). In the context of viral infection, siRNA1, siRNA2, and siRNA3 still maintained their efficacy in knocking down FASN protein expression (Fig. 6C). Compared to the scrambled siRNA control, FASN protein levels were reduced to approximately 43.60%, 55.43%, and 75.43% by these three individual siRNAs, respectively, in the context of virus infection (Fig. 6D). To explore the effects of FASN knockdown on viral productive infection, the expression of virion-associated proteins was detected via Western blot using an antibody produced by immunization of purified BoAHV-1 virions. As a result, a panel of bands indicative of distinct virion-associated proteins, denoted as “a,” “b,” and “c,” respectively, was readily detected by this antibody. The protein levels of all three bands were significantly decreased following transfection with either siRNA2 or siRNA3 (Fig. 6E). Specifically, compared to the scrambled siRNA control, the levels of band “a” were reduced to 45.74% and 31.09%, band “b” to 49.39% and 42.41%, and band “c” to 20.49% and 18.81% by siRNA2 and siRNA3, respectively (Fig. 6F). To further clarify how FASN knockdown affects viral productive infection, the mRNA levels of viral regulatory proteins bICP4 and bICP27, viral DNA polymerase, and viral glycoprotein gC, all of which are essential for viral productive infection, were examined in productively infected MDBK cells. Compared to the scrambled siRNA control, the levels of bICP4 decreased to 75.24% and 59.85%, bICP27 to 26.94% and 14.75%, viral DNA polymerase to 7.16% and 2.53%, and gC to 31.46% and 20.86% by siRNA2 and siRNA3, respectively (Fig. 6G). The reduced mRNA levels of the detected viral genes further confirmed that FASN protein knockdown decreases viral replication.

**Fig 7:**
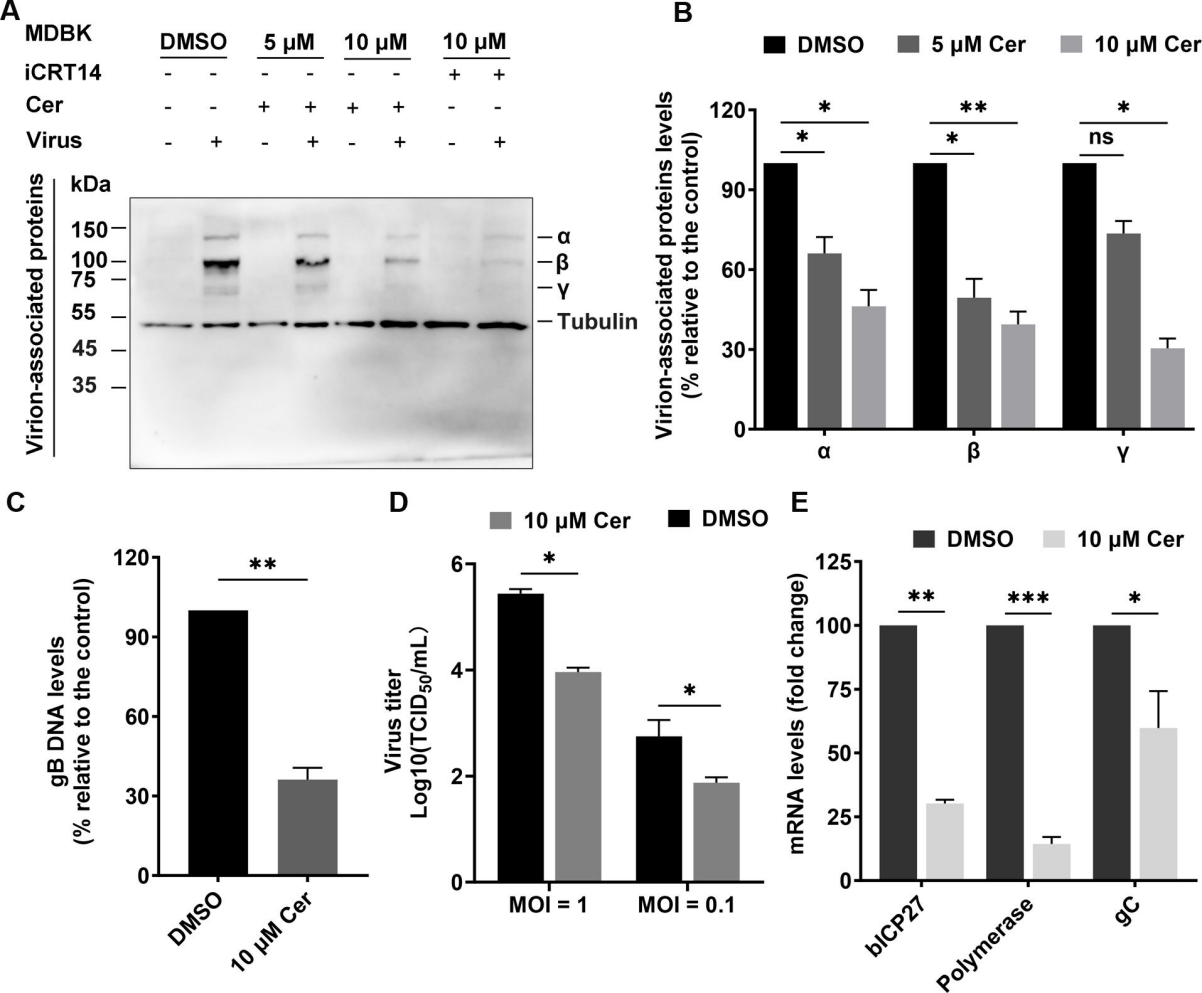
Determine the roles of FASN in BoAHV-1 productive infection in MDBK cells using a chemical inhibitor. (**A and C**) MDBK cells in six-well plates were infected with BoAHV-1 (MOI = 1) and treated either with DMSO control or with the FASN-specific inhibitor Cerulenin at the indicated concentrations. At 24 hpi, the cells were collected either to prepare cell lysates for Western blotting using an antibody against virus-associated proteins (VMRD, cat# P170703-001, 1:5,000) (**A**) or to extract DNA for subsequent qPCR analysis using gB-specific primers (**C**). (**B**) The intensity of bands for virus-associated proteins was quantified using the freeware software Image J. Changes in the levels of individual bands upon treatment with the inhibitor were calculated relative to those of the DMSO control, which was set at 100%. (**D**) Virus titers in the supernatants were determined and expressed as TCID_50_/mL. (**E**) MDBK cells in six-well plates were infected with the virus at an MOI of 1 for 24 h and treated with either DMSO control or 10 µM Cerulenin. Total RNA was then extracted from the cells for the detection of viral mRNA using RT-qPCR. Primers specific for bICP27, viral DNA polymerase, and viral protein gC were used, respectively. The data shown are means of three independent experiments, with error bars representing standard deviations. Significance was assessed by a standard *t*-test (ns, not significant; *P < 0.05; **P  <  0.01; ***P  <  0.001).

Cerulenin is a widely used FASN-specific inhibitor that contains an epoxy group, which mediates the inhibition of the β-ketoacyl-reductase activity of FASN (23, 24). In this study, the effects of Cerulenin on BoAHV-1 productive infection in MDBK cell cultures were further investigated. To achieve this, virus-infected cell cultures were treated with 5 µM or 10 µM Cerulenin. Three distinct bands, labeled as “α,” “β,” and “γ,” were clearly detected with shorter exposure times. The intensity of all these bands was significantly reduced by treatment with either 5 µM or 10 µM Cerulenin compared to the DMSO control (Fig. 7A). Specifically, the levels of band “α” were reduced to 66.07% and 46.20%, band “β” to 49.46% and 39.41%, and band “γ” to 73.57% and 30.40% by 5 and 10 µM Cerulenin, respectively, compared to the DMSO control (Fig. 7B). iCRT14, a chemical inhibitor of β-catenin signaling that has been shown to inhibit BoAHV-1 productive infection in cell cultures (25), was used as a control. As expected, iCRT14 significantly reduced the protein levels of all the bands, further validating the inhibitory effects of Cerulenin on BoAHV-1 productive infection in cell cultures (25). Quantitative RT-PCR analysis revealed that the levels of viral genome were reduced to approximately 36.17% following treatment with 10 µM Cerulenin, respectively, compared to the DMSO-treated control (Fig. 7C). Consistent with these findings, the treatment of virus-infected cells with 10 µM Cerulenin led to a reduction of viral titers by approximately 1.48- and 0.88-log, when cells were infected with the virus at MOI of either 0.1 or 1, respectively (Fig. 7D). Furthermore, treatment with 10 µM Cerulenin reduced the mRNA levels of the viral regulatory protein bICP27, viral DNA polymerase, and viral glycoprotein gC to 30.20%, 14.36%, and 59.76%, respectively, compared to the DMSO control (Fig. 7E). Notably, 10 µM Cerulenin had no significant cytotoxic effects on MDBK cells, as demonstrated by the Trypan blue exclusion test (data not shown). This indicates that the observed reduction in virus yields was not due to the cytotoxicity of the inhibitor. Collectively, studies using both siRNAs and chemical inhibitors consistently support this conclusion that FASN plays a crucial role in BoAHV-1 productive infection in MDBK cells.

**Fig 8:**
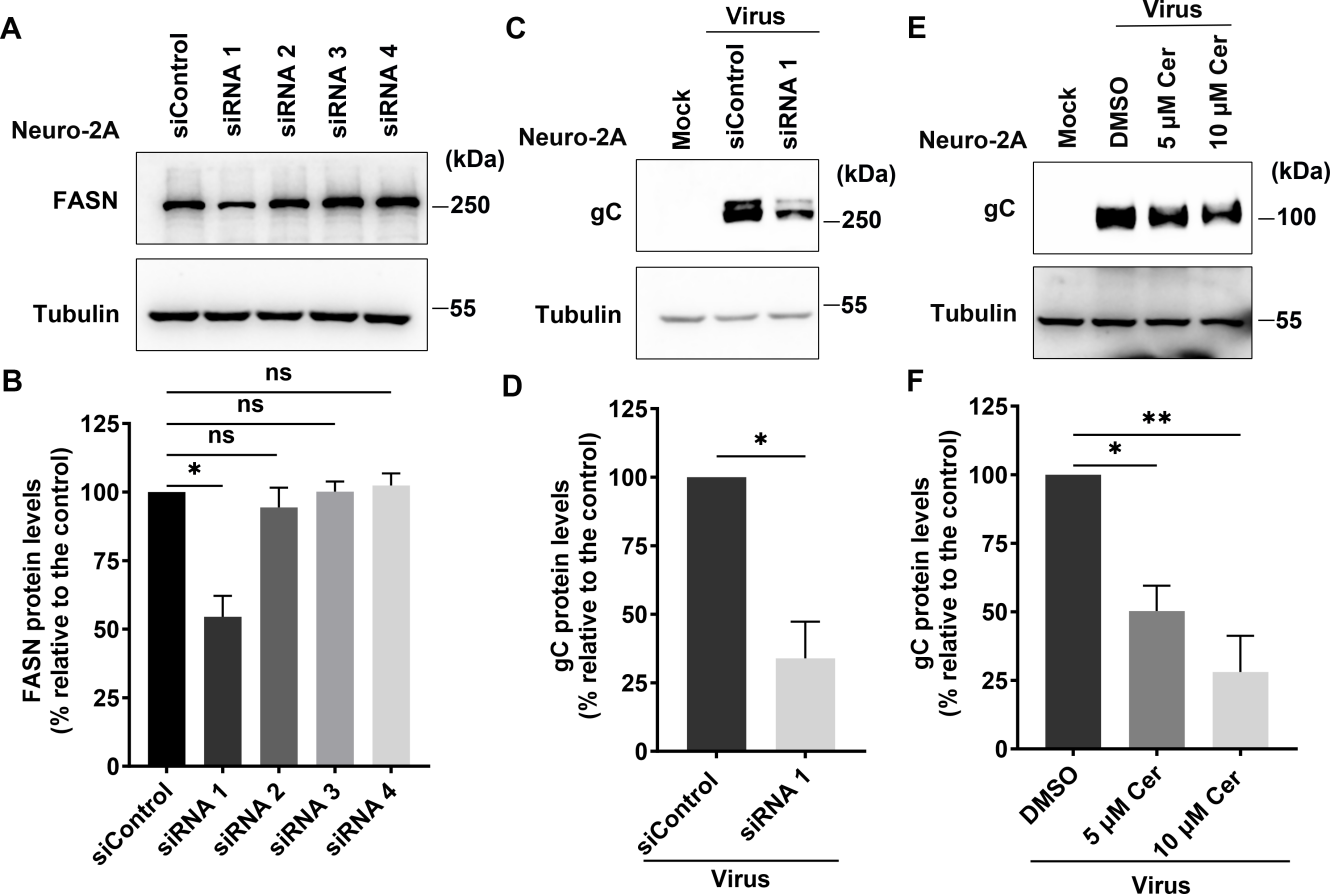
Determine the roles of FASN in BoAHV-1 productive infection in Neuro-2A cells using both siRNA and chemical inhibitor. (**A**) Neuro-2A cells in six-well plates were transfected with either scrambled siRNA (150 pmol) or individual siRNAs targeting FASN (150 pmol), referred to as siRNA1, siRNA2, siRNA3, and siRNA4, respectively. At 48 h post-transfection, cell lysates were prepared and subjected to Western blot analysis to detect FASN protein levels using an antibody against FASN (Proteintech, cat# 10624-2-AP, 1:10,000). (**C**) Neuro-2A cells in six-well plates were transfected with either scrambled siRNA (150 pmol) or siRNA1 (150 pmol). At 36 h post-transfection, the cells were infected with BoAHV-1 at an MOI of 10. After 24 h of infection, the cells were collected and subjected to Western blot analysis using a monoclonal antibody against viral protein gC (VMRD, cat#F2, 1:2,000). (**E**) Neuro-2A cells in six-well plates were infected with BoAHV-1 (MOI = 10) and treated either with DMSO control or with the FASN-specific inhibitor Cerulenin at the indicated concentrations. At 24 hpi, the cell lysates were prepared and subjected to Western blotting to detect viral protein gC. (**B, D, and F**) The band intensities were quantified using the free software Image J. The intensity of each band was first normalized to that of the respective loading control Tubulin, and then normalized to that of either DMSO-treated or scrambled siRNA-transfected control, which was arbitrarily set to 100%. The data shown are means of three independent experiments, with error bars representing standard deviations. Significance was assessed using a Student’s *t*-test (ns, not significant; *P < 0.05; **P  <  0.01).

Given that BoAHV-1 productive infection in Neuro-2A cells affects FASN protein expression, we further investigated whether FASN also plays a role in viral infection in the neural cell line using FASN knockdown and chemical inhibition. Among the four commercially available siRNAs (designated as siRNA1, siRNA2, siRNA3, and siRNA4), only siRNA1 effectively reduced FASN protein expression, decreasing it to 54.54% relative to that of the scrambled siRNA control (Fig. 8A and B). Transfection with siRNA1 significantly decreased the protein levels of viral glycoprotein gC (Fig. 8C). Compared to the scrambled siRNA control, the levels of gC protein were reduced to 33.91% by siRNA1 (Fig. 8D). The expression of viral protein gC was significantly reduced by 5 and 10 µM Cerulenin (Fig. 8E). Quantitative analysis indicated that the intensity of gC protein was reduced to 50.30% by 5 µM of Cerulenin and 28.00% by 10 µM Cerulenin, compared to the DMSO control (Fig. 8F).

### FASN-specific inhibitor, Cerulenin, traps viral protein gD in the Golgi apparatus

**Fig 9:**
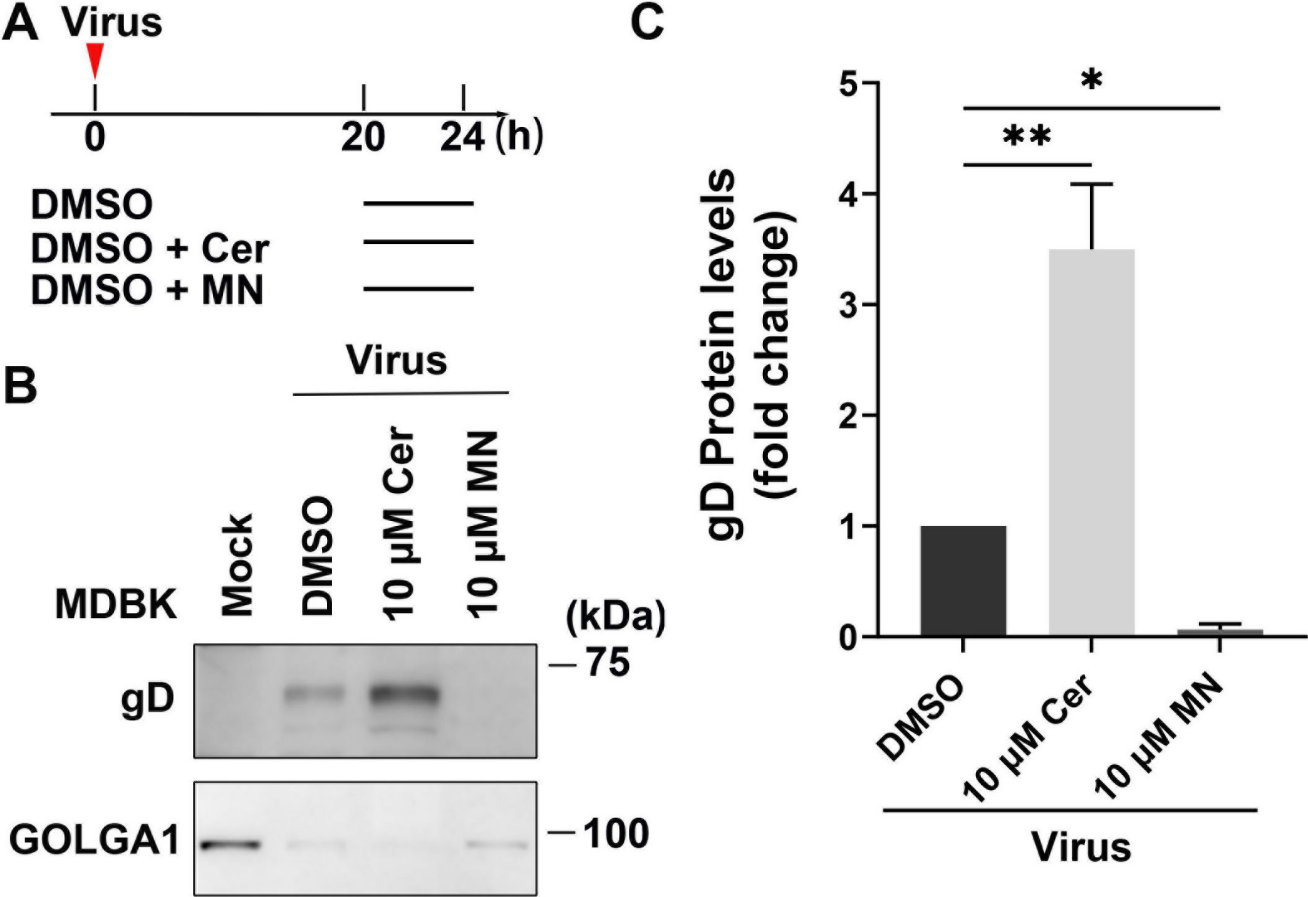
Determine the effects of FASN inhibitor, Cerulenin, on the accumulation of gD in the Golgi apparatus. (**A**) Diagram shows the treatment manner of virus-infected cells by both Cerulenin and Monensin. (**B**) MDBK cells of either mock-infected or virus-infected were treated either with vehicle control DMSO or with Cerulenin (10 µM) or Monensin (10 µM) for a duration of 4 h prior to the termination of infection. At 24 hpi, the cells were collected to purify the Golgi apparatus using a commercial purification kit (Beijing Biolabo Technology, cat# HR0247-50T). Subsequently, Western blot analysis was performed to detect the protein levels of gD in the Golgi fractions. GOLGA1, a marker of the Golgi apparatus, was used as a loading control and for subsequent quantitative analysis. (**C**) The band intensity was analyzed with the free software Image J. The intensity of each band was first normalized to that of the respective loading control GOLGA1, and then normalized to that of the control treated with DMSO, which was arbitrarily set to 1. The data shown are means of three independent experiments with error bars indicating standard deviations. Significance was assessed with a Student’s *t*-test (*P < 0.05, **P  <  0.01).

The Golgi is proposed as the key site for *de novo* envelopment of HSV-1/BoAHV-1 capsids and their subsequent egress to the plasma membrane (26–28). Since our findings indicate that partial FASN proteins located in the Golgi apparatus associate with viral protein gD (Fig. 3 to 5). This led us to hypothesize that the FASN signaling pathway may influence viral trafficking out of this organelle. To investigate this, we treated BoAHV-1-infected MDBK cells with Cerulenin at 20 hpi for a duration of 4 h. The cells were then collected at 24 hpi and subjected to Golgi isolation, as shown in the diagram (Fig. 9A). Monensin, a known chemical inhibitor of Golgi trafficking (29), was used as a control. When the isolated Gogi fractions were subjected to Western blot assay, we found that gD protein levels were significantly increased in the Golgi fractions by treatment with 10 µM of Cerulenin. Unexpectedly, treatment with Monensin resulted in a dramatic depletion of the viral protein gD in Golgi fractions (Fig. 9B). Quantitative analysis showed that gD protein levels increased to approximately 3.50-fold by 10 µM of Cerulenin, decreased to 7% by 10 µM of Monensin in comparison to the DMSO-treated control (Fig. 9C). Of note, two bands corresponding to the gD protein were detected using the gD-specific monoclonal antibody (VMRD, cat# 1B8-F11), as previously reported (30). These bands likely represent different glycosylation states of the protein. Collectively, these data suggest that Cerulenin treatment leads to the accumulation of gD, indicative of virions, in the Golgi apparatus. Therefore, FASN may play a crucial role in viral trafficking out of the Golgi apparatus.

## DISCUSSION

To date, manipulation of FASN protein expression for efficient replication has been reported in numerous viruses in the following way. Infections with CSFV, BVDV, and HCV have been shown to enhance FASN expression (31–33). FASN levels do not significantly change during DENV infection (34). Here, our studies suggest that BoAHV-1 either acute infection in cattle trigeminal ganglia (TG) neurons or productive infection in cell cultures, including MDBK and Neuron-2A cells, leads to a unanimous decrease of FASN protein expression (Fig. 1 and 2). Though BoAHV-1 productive infection leads to depletion of FASN, FASN still plays an important role in viral productive infection, as demonstrated using both siRNAs and chemical inhibitors (Fig. 6 to 8). Thus, FASN steady-state protein expression may be differentially affected by distinct viruses in virus-specific manners. Of note, BoAHV-1 productive infection induces host shut-off effects, and viral products such as the virus-encoded bICP0 protein are capable of mediating protein degradation (35). Therefore, elucidating the mechanisms underlying FASN depletion in virus-infected cells deserves further study in the future, which would significantly contribute to our comprehensive understanding of the detailed interplay between BoAHV-1 productive infection and FASN signaling. Moreover, the increased nuclear accumulation of FASN protein in a subset of latently infected TG neurons suggests that FASN may play a role in regulating viral latency. This intriguing possibility warrants further investigation in future studies.

FASN has been observed to have impacts on the infection of several viruses. For example, FASN participates in the formation of the CSFV replication complex, which is associated with the endoplasmic reticulum, through interaction with the viral protein (nonstructural protein 4B) NS4B, involving Rab18 protein (32). Similarly, Dengue virus protein NS3 redistributes FASN protein to the sites of viral replication to facilitate virus replication (34, 36). It seems that the localization of FASN to viral replication sites or replication complexes may represent a mechanism to facilitate virus infection observed in several viruses. Here, we found that the viral productive infection at later stages leads to re-localization of FASN protein, as demonstrated by making FASN puncta formed as detected under confocal microscope, and a portion of FASN protein located in the Golgi apparatus (Fig. 3 to 5). Since the virus productive infection leads to re-localization of the Golgi apparatus (22), it is no wonder that the Golgi harboring FASN will show a distinct distribution profile in response to viral infection. Of note, Golgi apparatuses are important sites for the viral replication cycle. We have previously reported that p-PLC-γ1(S1248) plays an important role in facilitating the viral trafficking from Golgi to the cell membrane (22). In the current study, we found that a subset of FASN protein co-localizes with the viral protein gD and virion-associated protein as detected in MDBK and Neuro-2A cells, respectively (Fig. 3 and 4). Importantly, we observed that a 4 h treatment with Cerulenin before termination of the infection process led to higher levels of accumulation of viral protein gD in the Golgi apparatus (Fig. 9). Therefore, FASN may play a crucial role in viral trafficking out of the Golgi apparatus, which could significantly expand our understanding of its role in viral infections within the virology community. Nevertheless, elucidating the detailed mechanisms by which FASN regulates viral trafficking out of the Golgi remains an intriguing area of study, deserving extensive investigation in the future. Surprisingly, treating virus-infected MDBK cells with Monensin, a known inhibitor of Golgi trafficking, led to a dramatic depletion of the viral protein gD in Golgi fractions (Fig. 9B). Future studies elucidating the mechanism underlying Monensin-induced gD depletion will be of great interest, as they may enhance our understanding of the virus replication process.

Importantly, FASN has been demonstrated to be critical for the replication or pathogenicity of a range of viruses, such as hepatitis C virus (HCV) (37), influenza virus (38), Epstein-Barr virus (EBV) (39, 40), and Chikungunya virus (CHIKV) (41). Of note, among herpesviruses, only EBV, a member of the gammaherpesvirus family, has been previously reported to interact with FASN. Here, we provide evidence that FASN also plays a significant role in the infection of BoAHV-1, a member of the alphaherpesvirus family.

In our study, we provide evidence showing FASN protein is potentially involved in BoAHV-1 lytic infection. A subset of the FASN protein was found to be co-localized with the viral protein gD, providing a possibility to affect virus infection. Moreover, for the first time, we identified that FASN may play a crucial role in viral trafficking out of the Golgi apparatus. These findings would contribute to our understanding of the mechanism of virus replication.

## MATERIALS AND METHODS

### Virus and cell cultures

MDBK cells were purchased from the Chinese model culture preservation center, Shanghai, China. Neuro-2A cells were kindly provided by Professor Dongli Pan from Zhejiang University. These cells were routinely maintained and passaged in DMEM supplemented with 10% fetal bovine serum (FBS). BoAHV-1 strain NJ-16-1, isolated from commercial bovine semen samples (42), was propagated in MDBK cells. Aliquots of virus stocks were stored at −70°C until use.

### Antibodies and reagents

FASN polyclonal antibody (pAb) (cat#10624-2-AP) and GP73 mouse monoclonal antibody (mAb) (cat# 66331-1-lg) were provided by Proteintech (Rosemont, IL, USA). FASN rabbit pAb (cat# A21182), β-Tubulin rabbit pAb (cat# AC015-200ul), GOLGA1 rabbit pAb (cat# A14688), and HRP-conjugated Donkey anti-goat IgG (H + L) (cat# AS031) were ordered from Abclonal Technology (Woburn, MA, USA). HRP-conjugated goat anti-mouse IgG (cat# BF03001) and HRP-labeled goat anti-rabbit IgG (cat# BF03008) were purchased from Biodragon (Wuhan, China). BoAHV-1 gD mouse mAb (cat# 1B8-F11), gC mouse mAb (cat# F2), and goat anti-IBR serum (cat# P170703-001) were purchased from VMRD (Puliman, WA, USA). Alexa FluorTM 488 goat anti-rabbit IgG (H + L) (cat# A11008), Alexa Fluor 647-conjugated goat pAb to rabbit IgG (cat# ab150079), and Alexa FluorTM 633 goat anti-mouse IgG (H + L) (cat# A21050) were provided by Invitrogen Life Technologies (Waltham, MA, USA). FASN-specific inhibitor Cerulenin (cat# HY-A0210) was ordered from MedChemExpress (Monmouth Junction, NJ, USA). Monensin (cat#S1753) was bought from Beyotime Biotechnology (Shanghai, China). FASN-specific siRNA was provided by Genepharma (Shanghai, China).

### Western blotting analysis

Cell lysates of either whole-cell extracts or cellular fractions of Golgi apparatus were prepared using RIPA lysis buffer (1 × PBS, 1% NP-40, 0.5% sodium deoxycholate, 0.1% SDS) supplemented with protease inhibitor cocktail. They were boiled in Laemmli sample buffer for 5 min, subsequently subjected to separation on SDS-PAGE (8% or 10%), and then transferred to polyvinylidene fluoride (PVDF) membranes. Immunoreactive bands were visualized using the Clarity Western ECL Substrate from NCM Biotech (cat# P10300).

For the designated studies, the band intensity was quantitatively analyzed with the free software Image J program (https://imagej.nih.gov/ij/download.html, accessed on 1 December 2020). Significance was assessed with a student’s *t*-test by using GraphPad Prism software (v8.0). *P* values of less than 0.05 (*P < 0.05) were considered significant for all the calculations.

### Immunofluorescence assay

MDBK cells in 24-well chamber slides (Nunc Inc., IL, USA) were either mock infected or infected with BoAHV-1 (MOI = 1). After infection for indicated time lengths such as 4, 16, and 24 hours (h), cells were fixed with 4% paraformaldehyde in PBS for 10 min at room temperature, permeabilized with 0.25% Triton X-100 in PBS for 10 min at room temperature, and blocked with 1% BSA in PBST for 1 h followed by incubation with indicated antibody in 1% BSA in PBS overnight at 4°C. After three washings, cells were incubated with secondary antibody labeled with distinct fluorescent dyes for 1 h in the dark at room temperature. After three washings with PBS, nuclei were stained with DAPI (4′,6-diamidino-2-phenylindole). Slides were covered with coverslips by using an antifade mounting medium (Electron Microscopy Sciences, cat# 50-247-04). Images were captured using a confocal microscope (Zeiss).

### siRNA transfection assay and virus infection assay

To screen the efficacy of the designated siRNAs, MDBK cells in six-well plates were transfected with scrambled siRNA or three FASN-specific siRNA of 150  pmol, provided by Genepharma (Shanghai, China). At 48 h post-transfection, cell lysates were prepared by using cell lysis buffer as described above and subjected to Western blot to detect the protein levels of FASN.

To determine the effects of siRNAs on BoAHV-1 infection, MDBK cells in six-well plates were transfected with scrambled siRNA or three FASN-specific siRNA of 150  pmol, provided by Genepharma (Shanghai, China). At 36 h post-transfection, the cells were infected with BoAHV-1 (MOI  =  1) for 24 h. After infection for 24 h, the cell lysates were prepared and subjected to Western blotting to detect the protein expression of Virion-associated protein using a commercial antibody against purified virions.

### Cerulenin treatment of MDBK cells during virus infection

MDBK cells that were confluent in 24-well plates were infected with BoAHV-1 (MOI  =  1) along with the treatment of chemical Cerulenin (MCE, cat# HY-A0210) at the indicated concentration for 1 h at 37°C. After three washes with PBS, a fresh medium with Cerulenin at the indicated concentrations was added to each well. After infection for 24 h, viral yields were titrated in MDBK cells, respectively. Cell cultures treated with DMSO were used as a control. The results were expressed as TCID_50_/mL calculated using the Reed-Muench formula (43).

### Quantification of viral genome by quantitative PCR

MDBK cells that were confluent in 6-well plates were infected with BoAHV-1 at an MOI of 1 for 24 h, along with treatment with either DMSO control or 10 µM Cerulenin. Then genomic DNA was extracted using a DNA extraction kit (Tiangen, DP304), following the manufacturer’s protocols. The purified DNA served as templates for qPCR to measure viral genomic levels using gene-specific primers as previously reported (44, 45). The primer sequences used were as follows: gB (forward reverse primer 5′- TGTGGACCTAAACCTCACGGT-3′ and reverse primer 5′-GTAGTCGAGCAGACCCGTGTC-3′), glyceraldehyde-3-phosphate dehydrogenase (GAPDH) (forward primer: 5′ CCATGGAGAAGGCTGGGG-3′ and reverse primer: 5′ AAGTTGTCATGGATGACC-3′). The analysis of GAPDH levels served as an internal control and subsequent normalization of gene expression. qPCR was performed on the ABI 7500 fast real-time system (Applied Biosystems, CA). The data were analyzed using the 2^−ΔΔCT^ method.

### Quantification RT-PCR assay

Total RNA was extracted using TRIzol LS Reagent (Ambion, Cat: 10296010) according to the manufacturer’s protocol. One microgram of freshly prepared RNA was used as a template for the synthesis of first-strand cDNA with commercial random hexamer primers for viral mRNA detection, employing the Thermoscript RT-PCR system Kit (Invitrogen, catalog #11146-024). The cDNA products served as templates for qPCR to measure FASN or viral mRNA levels using gene-specific primers as in previous studies (44, 46, 47). The primer sequences used were as follows: FASN (forward reverse primer 5′-ATTGTGGGC GGGATCAACCT-3′, reverse primer 5′-CGGCAATACCCGTTCCCTGA-3′). viral DNA polymerase (forward reverse primer 5’-GCGAGTACTGCATCCAAGAT-3′ and reverse primer 5’-AATCTGCTGCCCGTCAAA-3′). bICP4 (forward reverse primer 5′- GCCACAGCTCGTTCATCAC-3′ and reverse primer 5′-GCTTCTGGTCGCAGTCGTAG-3′), bICP27 (forward reverse primer 5′- AAACCTGGTAGACGCACTGG-3′ and reverse primer 5′-ACGATAGGGTCTTTGGTGCG-3′), gC (forward reverse primer 5′-ACTATATTTTCCCTTCGCCCG-3′ and reverse primer 5′-TGTGACTTGGTGCCCATG-3′), and 18sRNA (forward reverse primer 5′- GTAACCCGTTGAACCCCATT-3′, and 5′-CCATCCAATCGGTAGTAGCG-3′). The analysis of 18sRNA mRNA served as an internal control. qPCR was performed on the ABI 7500 fast real-time system (Applied Biosystems, CA). Separate amplification of GAPDH was used to normalize gene expression. The data were analyzed using the 2^−ΔΔCT^ method.

### Infection of calves

Four-month-old female Holstein cows, which had not been vaccinated against BoAHV-1 and were antibody-negative for BoAHV-1 (as determined using a commercial BoAHV-1 IgG indirect ELISA kit; BioStone Animal Health, Southlake, TX, USA; catalog number 10074-05), were used in these studies. Female cows were usually used for BoAHV-1 infection studies. This preference can be partly explained by the fact that females generally mount a stronger immune response to viral infections compared to males. Thus, female animals are more likely to survive (48, 49), which is advantageous for the study. In addition, female animals often have a more consistent hormonal environment during certain stages of their life cycle, which can reduce variability in experimental results (50, 51). Particularly, BoAHV-1 productive infection and latency can be differentially affected by hormones, such as progesterone (52). The calves were inoculated in each nostril and eye with 1 mL of DMEM containing 1 × 10^7^ PFU of virus, without scarification, for a total of 4 × 10^7^ PFU per animal, as previously described (53). The calves were maintained under strict isolation conditions and were administered antibiotics before and after BoAHV-1 infection to prevent secondary bacterial infections, as described elsewhere (54). The time point of acute infection and latent infection was selected as 4 and 60 days post-infection, as previously described (53). Following euthanasia, the TG tissues were collected, sectioned into small pieces, and processed according to standard histopathological protocols, including formalin fixation and paraffin embedding.

Animal use was approved by the Institutional Animal Care and Use Committee of Hebei University (Approval number 402,106,003) and conducted in accordance with the Guide for the Care and Use of Laboratory Animals by the National Research Council.

### IHC assay

TG tissues embedded in paraffin were cut into thin sections (10 µm), mounted on glass slides, and processed for IHC following standard protocols as described elsewhere (55). The slides were incubated overnight at 4°C in a humidified chamber with antibodies against FASN (Proteintech, cat#10624-2-AP, diluted 1:500 in 1% BSA in PBST [PBS + 0.1% Tween-20]). Then the slides were washed in 1 × PBS and incubated in biotinylated goat anti-rabbit IgG (Vector Laboratories, cat# PK-6101) for 30 min at room temperature in a humidified chamber. Avidin-biotinylated enzyme complex was applied to the slides for 30 minutes of incubation at room temperature. After three washes in 1 × TBS, the slides were incubated with freshly prepared substrate (Vector Laboratories, cat# SK-4800), rinsed with distilled water, and counterstained with hematoxylin. Images were captured under a light microscope (Nikon, Tokyo, Japan).
